# Study on the Rolling Forming Process of Mg/Al Composite Foils with Low Edge Cracking

**DOI:** 10.3390/ma19040694

**Published:** 2026-02-11

**Authors:** Guang Feng, Zhaopeng Li, Ning Wang, Zhongxiang Li, Shaoyong Du

**Affiliations:** 1 College of Mechanical Engineering, Taiyuan University of Technology, Taiyuan 030024, China; 17736987054@163.com (Z.L.); wn13694117629@163.com (N.W.); 2023510091@link.tyut.edu.cn (Z.L.); du_shyog@126.com (S.D.); 2 Engineering Research Center of Advanced Metal Composites Forming Technology and Equipment, Ministry of Education, Taiyuan 030024, China; 3 TYUT-UOW Joint Research Centre of Advanced Forming and Manufacturing Technology, Taiyuan 030024, China

**Keywords:** Mg/Al composite foil, rolling temperature, reduction ratio, edge cracking

## Abstract

**Highlights:**

**What are the main findings?**

- A rolling temperature of 400 °C and a reduction ratio of 35% are the optimal simulated process parameters for achieving superior Mg/Al composite interface bonding
- The initial 0.5 mm thick Mg/Al plates were successfully rolled down to 30 μm via a 21-pass process consisting of “hot rolling bonding + hot rolling + cold rolling and annealing”.
- This multi-stage rolling process can effectively inhibit the initiation and propagation of edge crackings in the composite foils.

**What are the implications of the main findings?**

- A feasible multi-pass rolling process scheme is provided for the preparation of ultra-thin Mg/Al composite foils with low edge cracking.
- It is revealed that the edge cracking issue of composite foils can be significantly controlled by regulating temperature, reduction ratio and annealing treatment.
- The research results offer important process references for the industrial-scale preparation of high-performance Mg/Al composite foils.

**Abstract:**

Edge cracking is prone to occur during the rolling of Mg/Al composite foils. Herein, a hybrid hot–cold rolling process was adopted to fabricate 30 μm thick Mg/Al composite foils with low edge cracking. AZ31B magnesium alloy and 5052 aluminum alloy sheets, both with an initial thickness of 0.5 mm, were chosen as research materials. Numerical simulations of composite pass were conducted at 300–450 °C with reduction ratios of 25–40%, and the optimal parameters were identified as 400 °C and a 35% reduction ratio. Based on this, multi-pass rolling experiments were performed: composite pass was heated at 400 °C for 10 min with 35% reduction ratio, hot rolling passes at 300 °C for 1–3 min, and subsequent cold rolling with 15% reduction ratio. After 21 rolling passes, 30 μm thick Mg/Al composite foils with low edge cracking were successfully prepared. Interface and metallographic characterizations demonstrated that the diffusion layer thickness varied slightly during hot rolling and increased moderately during cold rolling. For the magnesium alloy, hot rolling improved microstructural uniformity and reduced shear bands, while cold rolling caused decreased uniformity and the gradual emergence of shear bands. The research results provide a reference for the preparation of high-quality Mg/Al composite foils.

## 1. Introduction

Magnesium alloys are characterized by low density, high specific strength and high specific stiffness [1,2,3]; aluminum alloys, in contrast, possess excellent formability and superior corrosion resistance [4]. Mg/Al composite materials fabricated by specific composite using these two dissimilar materials integrate the respective advantages of both constituents, resulting in a novel lightweight material with high strength and excellent corrosion resistance. Such composites hold broad engineering application prospects in the fields of electronic communication, aerospace, national defense, military industry, etc. [5,6].

In order to obtain metal composite foils with stable performance, researchers have developed a variety of manufacturing processes. Among these, the roll bonding method has emerged as the most widely used technique for producing metal composite foils to date, owing to its advantages, including simplicity of operation, cost-effectiveness, and high efficiency [7,8,9]. At present, the combination process of “bonding first, then thinning” is generally adopted in preparation of metal composite foils; that is, dissimilar metal composite sheets and strips are first obtained via thin sheet roll bonding, and then a series of thinning processes are applied to finally produce dissimilar metal composite foils. However, in the practical rolling production of Mg/Al composite materials, the poor plasticity of magnesium alloys makes the materials highly prone to edge cracking [10,11]. Aiming at the tough problem of edge cracking during the rolling of Mg/Al composite materials, numerous scholars have carried out relevant research and achieved important progress. Jia et al. [12] investigated the edge cracking behavior of AZ31 magnesium alloy during hot rolling (250–400 °C, reduction ratios 30–45%) using finite element simulation and experimental tests, and established a reliable theoretical basis for edge cracking prediction based on the optimized Freudenthal fracture criterion to predict the initiation and propagation of cracks during hot rolling. The results indicated that the dominant fracture mode of edge cracking was 45° shear cracking, and the crack depth increased with the decrease in rolling temperature and the increase in reduction ratio. Zhao et al. [13] modified the Lemaître damage model based on the damage prediction mechanism under the generalized stress states to mitigate edge cracking in Mg/Al composite sheets, which was applied to analyze the key mechanical factors governing the formation of edge cracking; on this basis, they proposed an embedded rolling bonding technology. The results showed that compared with the conventional process, this technology reduced the maximum edge stress triaxiality of the sheets from −0.02 to −1.56, which significantly enhanced the triaxial compressive stress effect at the edges, thereby weakening the nucleation and growth of microvoids and further inhibiting the occurrence of edge cracking. Tian et al. [14] found that conventional single-pass rolling with a large reduction of magnesium alloy tended to cause edge cracking, while width-restricted rolling could produce crack-free sheets. They explored the evolution laws of the microstructure of sheets under the two rolling methods, and elucidated the rules that the development of twinning in conventional rolling easily induces cracking, and dislocation pile-up can hinder crack propagation, whereas the associated deformation mechanism of dislocation shear twinning can effectively inhibit cracking. Kamran et al. [15] studied the effect of the multi-pass rolling process on the edge cracking of AZ31 magnesium alloy. The results showed that at a rolling temperature of 250–350 °C, a total reduction ratio of 85% without cracking was achieved after 26 rolling passes, which significantly improved the formability of magnesium alloy; however, severe edge cracking occurred when the reduction ratio increased to 90%, demonstrating that the pass schedule is crucial for the edge cracking behavior of magnesium alloy during multi-pass rolling. Although existing studies have provided valuable references for edge cracking control of pure magnesium alloys and Mg/Al composite materials, there remain certain limitations currently: most relevant studies focus on thick plates or pure magnesium alloy materials, while relatively few studies have been conducted on Mg/Al composite foils with a thickness of 20–50 μm, and the rolling processes adapted to such thickness specifications of composite foils are far from being optimized.

Based on this, a multi-pass rolling process was adopted to fabricate Mg/Al composite foils in this study, and two key process parameters (rolling temperature and reduction ratio) were precisely controlled to inhibit the occurrence of edge cracking during the rolling of Mg/Al composite foils. First, the ABAQUS 2022 numerical simulation software was employed to analyze the stress state and edge cracking characteristics during the composite pass rolling process, thereby obtaining the optimal rolling parameters for the composite pass. Subsequently, rolling experiments were conducted. After achieving rolling composite by adopting the optimal composite pass process parameters derived from finite element analysis, multi-pass “hot rolling + cold rolling” thinning experiments were carried out, and the rolling process for preparing 30 μm thick Mg/Al composite foils was developed. This work provides theoretical and experimental foundations for the fabrication of high-quality Mg/Al composite foils.

## 2. Materials and Methods

The raw materials selected for the experiments were AZ31B magnesium alloy sheets and 5052 aluminum alloy sheets, all with an initial dimension of 150 mm (length) × 70 mm (width) × 0.5 mm (thickness). Their chemical compositions are given in Table 1.

### 2.1. Numerical Simulation Method

**Figure 1:**
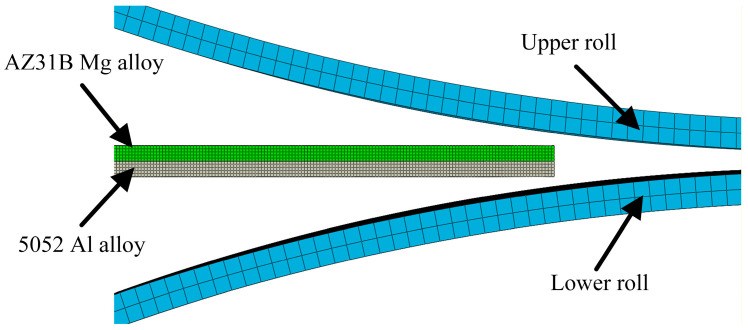
Finite element analysis model for rolling Mg/Al composite foil.

**Figure 2:**
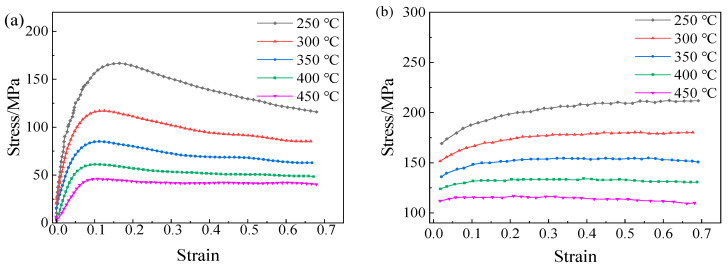
Material Properties of 5052 Aluminum Alloy and AZ31B Magnesium Alloy: (**a**) Mg alloy; (**b**) Al alloy.

In this study, primary efforts were devoted to the analysis of edge stress state and damage of Mg/Al composite foils under the combined action of rolling force and thermal effect. The ABAQUS software was employed to perform thermos-mechanically coupled explicit dynamic analysis on the rolling process of Mg/Al composite foils, and the established finite element analysis model is shown in Figure 1. To ensure the stability and accuracy of the finite element calculation, the magnesium and aluminum sheets were defined as elastoplastic bodies, while the upper and lower rolls were set as rigid bodies. The penalty contact method was adopted for the contact between the rolls and the foil; the friction coefficients between the rolls and the magnesium sheet, the rolls and the aluminum sheet were set to 0.4 and 0.35, respectively, and the friction coefficient between the aluminum sheet and the magnesium sheet was set to 0.3 [16]. The ambient temperature and the initial temperature of the rolls were set to 20 °C, the convective heat transfer coefficient between the slab and air was set to 0.016 mW/(mm^2^·°C), and the contact heat transfer coefficient between the rolls and the upper and lower metal slabs was 11 mW/(mm^2^·°C). The rolling speed was fixed at 0.12 m/s. Four rolling temperatures (300 °C, 350 °C, 400 °C, 450 °C) and four rolling reduction ratios (25%, 30%, 35%, 40%) were designed for the analysis. The material properties of 5052 aluminum alloy and AZ31B magnesium alloy were defined based on Figure 2, with their stress–strain curves input into the material database of the ABAQUS software. For mesh generation, the C3D8RT element type was adopted for both magnesium and aluminum alloys with a mesh size of 0.1 mm, and five mesh layers were divided in the thickness direction for each alloy sheet. In addition, the Cockcroft & Latham criterion was incorporated into the simulation calculation process to establish a correlation between the stress and strain data at the foil edges and the edge cracking state, which serves as the critical criterion for predicting edge cracking in magnesium alloy materials. Through the above analyses, the forming process parameters for rolling Mg/Al composite foils with low edge cracking were obtained.

### 2.2. Experimental Methods

**Figure 3:**
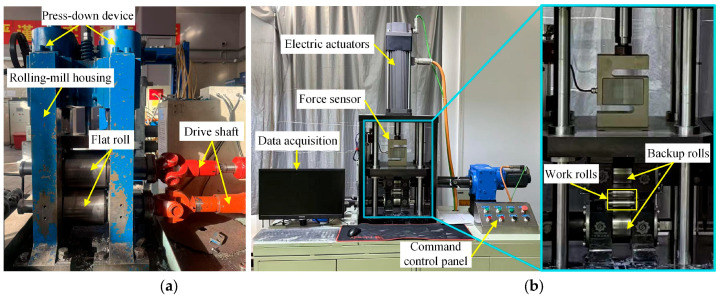
Rolling experiment equipment for Mg/Al composite foil: (**a**) two-high rolling mill; (**b**) precision four-high rolling mill.

During the experiments, the to-be-bonded surfaces of the raw materials were first ground to remove the surface oxide layer, and the two ends of the thin sheets were bound with aluminum wires after stacking and slab assembly. A self-designed two-high rolling mill by our research group was employed for hot rolling, with a roll diameter of 150 mm and a rolling speed set to 0.12 m/s, as shown in Figure 3a. Cold rolling was performed on a four-high rolling mill designed by our research group, with a roll diameter of 22 mm and barrel length of 44 mm, as shown in Figure 3b. Given the complexity of the hot rolling composite pass experiment, the optimal combination of rolling temperature and reduction ratio parameters was determined via finite element simulation prior to the formal experiments. Subsequently, rolling experiments were conducted with the optimal rolling parameters obtained from the simulation. Prior to the hot rolling composite pass, the billets were placed in a tube furnace (Dongguan Zhiyuan High-Temperature Machinery Technology Co., Ltd., Dongguan, China) for heating and heat preservation for 10 min, and argon gas was introduced into the furnace to prevent the ground surfaces from re-oxidation. After the heat preservation, the specimens were quickly taken out and fed into the two-high rolling mill for composite pass rolling.

Upon the completion of composite bonding, the rolling temperatures for the hot rolling thinning passes were set to 250 °C, 300 °C and 350 °C to screen out the optimal thinning temperature. Thinning experiments were then carried out at this optimal temperature to further determine the appropriate pass reduction ratio. When the foil thickness was reduced to the minimum rollable thickness of the two-high rolling mill, a precision four-high rolling mill was adopted for subsequent cold rolling thinning, which effectively lowered the minimum rollable thickness of the foil. Owing to the small roll width of the precision four-high rolling mill, the Mg/Al composite foils were cut into smaller sizes before cold rolling: the foils were trimmed into specimens with dimensions of 100 mm (length) × 15 mm (width), and the rolling experiments were performed after short-time annealing of these specimens.

After the entire rolling process, sampling was conducted on the Mg/Al composite foils. The micro-morphology of the composite foils was observed using a JEOL-IT500 Scanning Electron Microscope (Japan Electron Optics Laboratory Co., Ltd., Tokyo, Japan), abbreviated as SEM, and the metallographic structures of the foils were examined with a Leica DM4M optical microscope (Leica Microsystems GmbH, Wetzlar, Germany).

## 3. Results and Discussion

### 3.1. Composite Pass Rolling Simulation of Mg/Al Composite Foils

Due to the poor plasticity of magnesium alloys and excellent plasticity of aluminum alloys, edge cracking primarily initiates on the magnesium alloy side during rolling. If edge cracking occurs on the Al side, it must have already initiated on the Mg side beforehand. Therefore, this study focuses on the research related to edge cracking on the Mg side.

**Figure 4:**
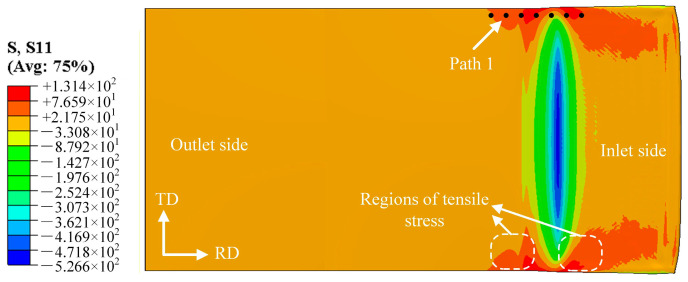
Stress distribution diagram along the RD within the rolling deformation zone.

In this study, the end of the rolled piece entering the rolling mill first is defined as the entry end, and the end entering subsequently is defined as the exit end. Figure 4 shows the stress distribution map along the rolling direction (RD) at the bonding interface on the magnesium alloy side during the composite pass, where positive values represent tensile stress and negative values represent compressive stress. The stress variation curve at the foil edge was extracted along Path 1, which is situated 0.5 mm from the foil edge with a spacing of 0.1 mm between adjacent measurement points. The length of Path 1 was determined based on the distribution range of stress peaks, aiming to fully characterize the section of the rolling deformation zone where the material bears the maximum stress. During the rolling of Mg/Al composite foils, the central part of the deformation zone is mainly subjected to compressive stress, while additional tensile stress is generated at the edge. This is because the central metal of the foil is constrained by the edge metal, which impedes its plastic flow, whereas the flow of the edge metal remains unrestricted. Consequently, the relatively high tensile stress at the edge can induce edge cracking [17], and the rolling temperature and reduction ratio directly affect the magnitude of the tensile stress at the edge.

**Figure 5:**
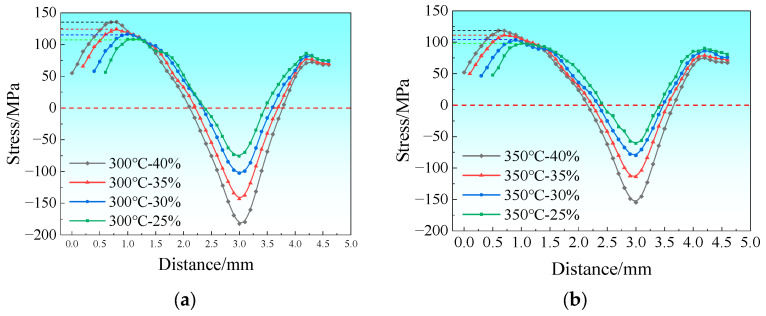

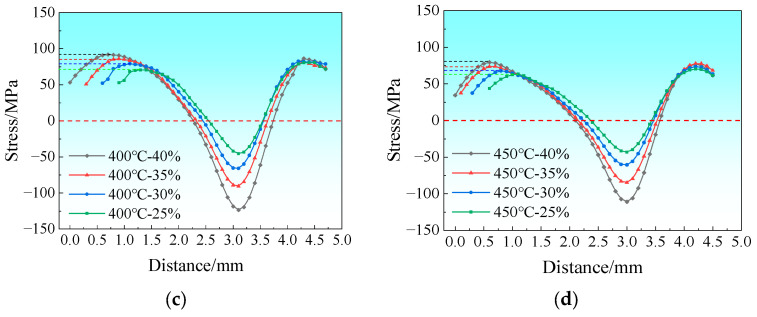
Stress distribution curve along the RD within the rolling deformation zone: (**a**) 300 °C; (**b**) 350 °C; (**c**) 400 °C; (**d**) 450 °C.

The RD stress variation curves of the rolling deformation zone along Path 1 are presented in Figure 5. The dashed line indicates the boundary at zero stress. Figure 5a depicts the stress variation curves at a rolling temperature of 300 °C with the reduction ratio ranging from 25% to 40%. As shown in the figure, the peak tensile stress increases from 108.3 MPa to 135.8 MPa with increasing reduction ratio, corresponding to a growth rate of 25.4%. Figure 5b shows the stress variation curves at a rolling temperature of 350 °C, where the tensile stress peak decreases significantly, with the maximum tensile stress being approximately 118.7 MPa. Figure 5c illustrates the stress variation curves at 400 °C, with the maximum tensile stress reaching about 91.9 MPa, and Figure 5d shows those at 450 °C with the maximum tensile stress of around 79.5 MPa.

In conclusion, the peak tensile stress at the edge of Mg/Al composite foils during rolling increases with the rise in reduction ratio and decreases with the increase in rolling temperature.

**Figure 6:**
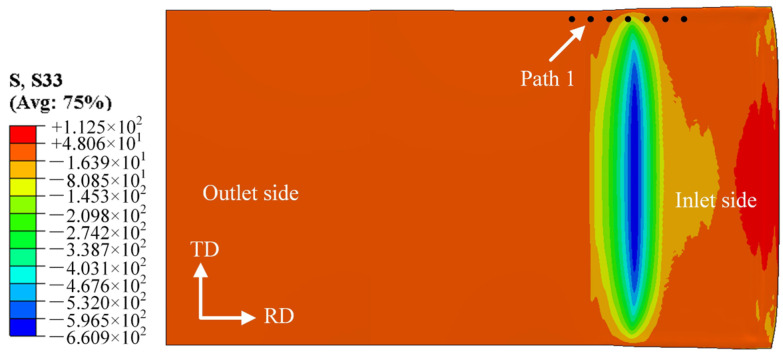
Stress distribution diagram along the TD within the rolling deformation zone.

**Figure 7:**
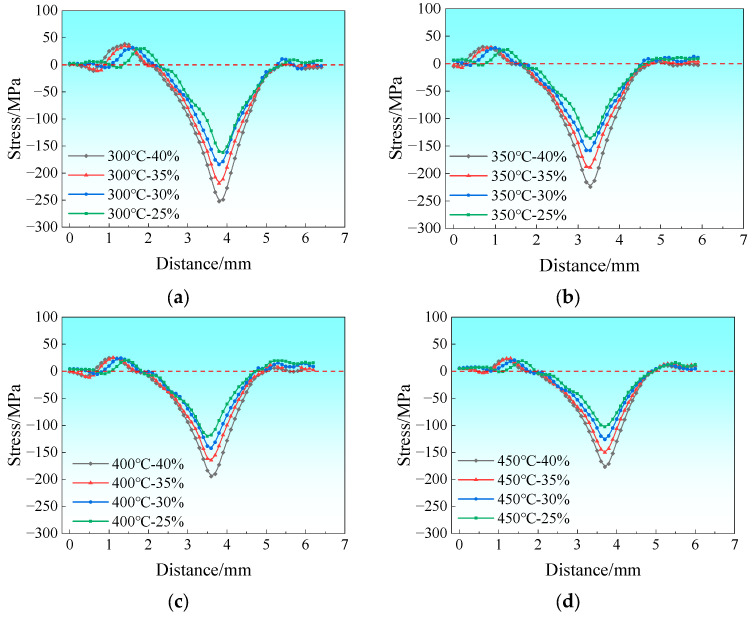
Stress distribution curve along the TD within the rolling deformation zone: (**a**) 300 °C; (**b**) 350 °C; (**c**) 400 °C; (**d**) 450 °C.

The stress distribution characteristics at the bonding interface on the magnesium alloy side along the transverse direction (TD) are illustrated in Figure 6. The dashed line stands for the dividing line where the stress is zero. Both compressive and tensile stresses coexist in the TD, with compressive stress dominating overall; moreover, the compressive stress increases gradually from the edge to the center of the deformation zone. The peak tensile stress at the edge rises with the decrease in rolling temperature and the increase in rolling reduction ratio. When the holding temperature is kept constant at 400 °C, the peak tensile stress increases from 20.3 MPa at a 25% reduction ratio to 25 MPa at a 40% reduction ratio, with a growth rate of only 18.8%. This indicates that the variation in reduction ratio exerts a limited effect on the magnitude of the peak tensile stress in the TD direction at this temperature. When the reduction ratio is kept constant at 35%, the peak tensile stress increases from 25 MPa at 400 °C to 34.8 MPa at 250 °C, representing a growth rate of 39.2%. In comparison with the peak tensile stress in the RD, however, the tensile stress in the TD direction has a minor effect on edge cracking, as shown in Figure 7.

**Figure 8:**
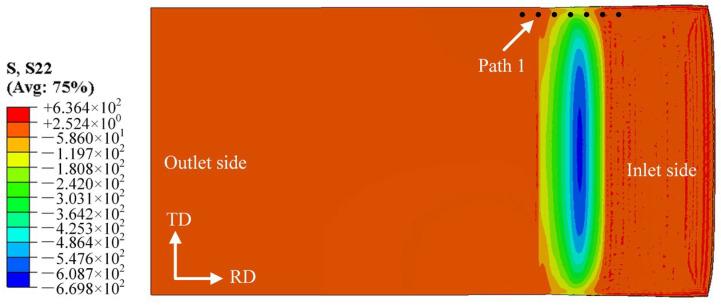
Stress distribution diagram along the ND within the rolling deformation zone.

**Figure 9:**
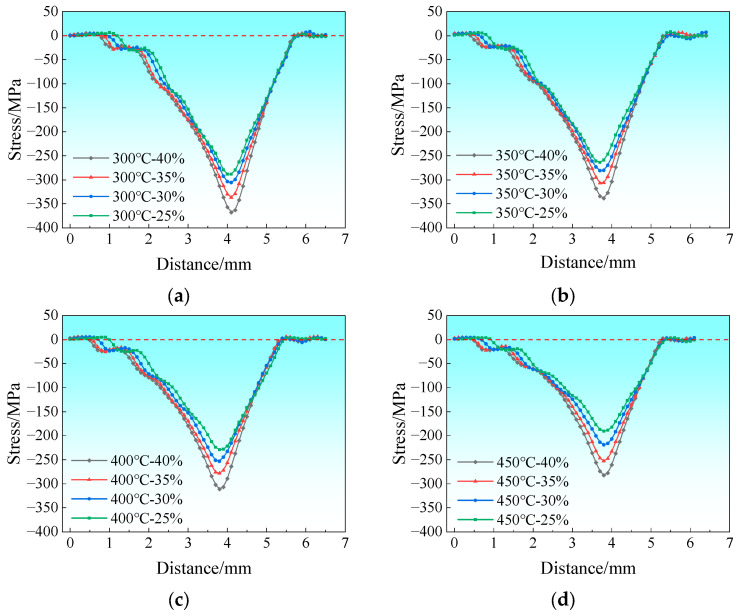
Stress distribution curve along the ND within the rolling deformation zone: (**a**) 300 °C; (**b**) 350 °C; (**c**) 400 °C; (**d**) 450 °C.

Figure 8 presents the stress distribution map of the rolling deformation zone along the normal direction (ND) at the bonding interface on the magnesium alloy side during the composite pass. Among them, the dashed line represents the zero-stress dividing line. Since the ND stress in the deformation zone is predominantly compressive during the rolling of Mg/Al composite foils, all extracted stress values are negative. In the subsequent analysis, all ND stress values are analyzed in terms of their absolute values. The simulation results show that the foil is mainly subjected to compressive stress in the ND, and the compressive stress increases gradually from the edge to the center of the foil; no significant tensile stress distribution is observed at the edge in the regions before and after the deformation zone. Under the conditions of different holding temperatures and rolling reduction ratios, the peak tensile stress values in the rolling deformation zone along the ND direction are all close to 0, as shown in the stress variation curves in Figure 9, indicating that rolling temperature and reduction ratio exert an insignificant effect on the peak tensile stress in this direction.

Cracks generated during the rolling of Mg/Al composite foils are mainly concentrated on the magnesium alloy side, and this phenomenon is primarily attributed to two reasons: firstly, the poor plasticity of magnesium alloys; secondly, the thin thickness of the composite foil combined with the low specific heat capacity and high emissivity of magnesium alloys accelerates the heat dissipation rate, resulting in a significant temperature drop of the foil [18]. In this study, based on the Cockcroft & Latham criterion, as shown in Equation (1), the edge damage value was calculated from the cumulative equivalent plastic strain and equivalent stress to analyze the edge cracking of Mg/Al composite foils prepared under the process parameters of different rolling temperatures and reduction ratios.

(1) $$C=\int_{0}^{{\bar{\varepsilon }}_{f}}\frac{\sigma_{1}}{\bar{\sigma }}d\bar{\varepsilon }$$

where *C* is the simulated edge damage value; ${\bar{\varepsilon }}_{f}$ is the equivalent plastic strain; $\sigma_{1}$ is the maximum tensile stress; $\bar{\sigma }$ is the equivalent stress; and *d*$\bar{\varepsilon }$ is the equivalent strain increment.

Ning et al. [19] established a critical fracture damage value prediction model for edge cracking based on the deformation activation energy of AZ31B Mg alloy. The specific calculation formulas are as follows:

(2) $$C_{f}=0.84439-0.01015\ln\left[\frac{2v\sqrt{\frac{H-h}{r}}}{H+h}\exp(\frac{2.47\times 10^{5}}{8.314\times T})\right]$$

where *C_f_* is the calculated critical fracture damage value; *v* is the rolling speed, *H* is the inlet thickness, *h* is the outlet thickness, *r* is the roll radius, and *T* is the rolling temperature.

Temperature data at different edge positions on the magnesium alloy side during entry into the rolling deformation zone was extracted from the simulation results, and the critical fracture damage value *C_f_* under the experimental conditions was calculated in combination with Equation (2). On this basis, the variation curves of *C_f_* with rolling distance under different rolling temperatures and reduction ratios were plotted. This parameter varies at different positions of the same foil, which is mainly attributed to the variation in the temperature field during the rolling process. To determine whether cracking occurs, a numerical simulation damage criterion was adopted in this study by comparing the simulated damage value *C* with the critical fracture damage value *C_f_*. If *C* > *C_f_*, it indicates that the internal damage accumulation of the magnesium alloy has exceeded the material’s bearing capacity limit, thereby inducing edge cracking; in contrast, if *C* ≤ *C_f_*, the material damage does not reach the critical state and no edge cracking occurs.

**Figure 10:**
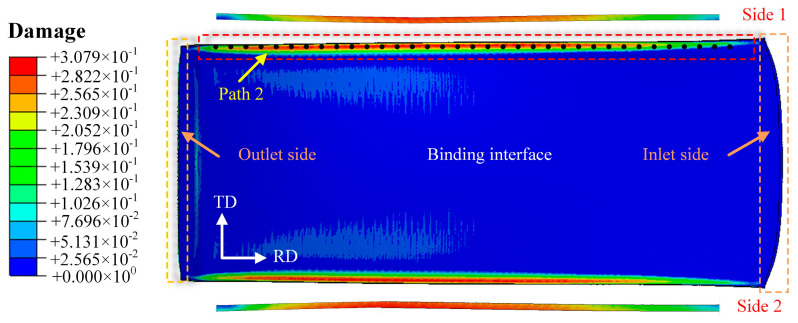
Damage distribution of Mg/Al composite foil interfacial zone.

**Figure 11:**
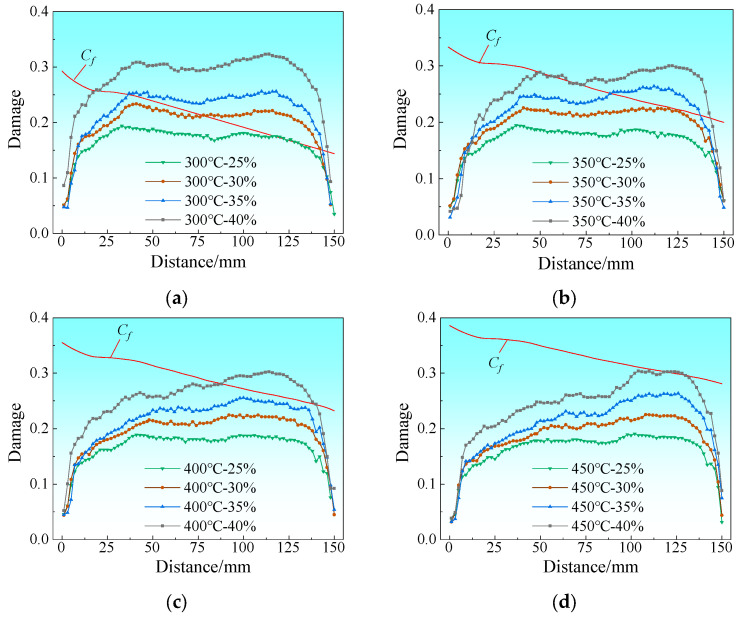
Distribution curve of damage value at the Mg side edge under different rolling reduction ratios: (**a**) 300 °C; (**b**) 350 °C; (**c**) 400 °C; (**d**) 450 °C.

Figure 10 shows the damage distribution map at the bonding interface on the magnesium alloy side of the Mg/Al composite foil. It can be seen from the figure that the maximum damage values are mainly concentrated in the edge region of the foil and decrease gradually from the edge to the center. This indicates that the edge is more likely to reach the critical fracture damage value of the material, which accelerates crack initiation and ultimately leads to edge cracking defects. Path 2 is defined as the extraction path for edge cracking damage values. Figure 11 presents the distribution curves of edge damage values under different process parameters. The *C_f_* values exhibit basically the same variation trend at all rolling temperatures: as the rolled piece gradually enters the roll gap from the entry end, the temperature drop rate is relatively high due to the increased contact area and the large temperature difference with the surrounding environment. As the rolled piece fully enters the roll gap, the heat generated by plastic deformation reduces the temperature drop rate to a certain extent.

The results show that at a rolling temperature of 300 °C with the reduction ratio ranging from 25% to 40%, there are consistent regions where *C* exceeds C_f_. This demonstrates that the magnesium alloy has poor plasticity at this temperature and is prone to edge cracking, as shown in Figure 11a. When the rolling temperature is increased to 350 °C, *C* does not exceed *C_f_* at reduction ratios of 25% and 30%; however, edge cracking initiates with a further increase in the rolling reduction ratio, as illustrated in Figure 11b. When the rolling temperature is raised to 400 °C, the peak tensile stress at the Mg alloy edge decreases to 91.9 MPa (Figure 5c). This is because higher temperatures improve the plasticity of Mg alloy by promoting dynamic recrystallization [20,21], reducing the stress concentration at the edge. Moreover, a 35% reduction ratio balances interface bonding (sufficient plastic deformation to remove oxide layers) and edge damage (avoiding excessive deformation beyond the plastic limit), thus making 400 °C and 35% the optimal parameters. At a rolling temperature of 450 °C, when the reduction ratio exceeds 40%, the degree of plastic deformation of the material has gone beyond its plastic deformation limit, resulting in the occurrence of edge cracking at this reduction ratio, as shown in Figure 11d. Nevertheless, when the reduction ratio is increased to over 40%, the degree of plastic deformation of the material rises beyond its plastic deformation limit, resulting in edge cracking at this reduction ratio, as shown in Figure 11c,d.

Considering the process requirements of achieving the maximum thinning rate, reducing the number of rolling passes and lowering energy consumption comprehensively, the optimal process parameters for the composite rolling pass in this experiment are determined as follows: a rolling temperature of 400 °C and a reduction ratio of 35%. Under all rolling parameter conditions, the edge cracking regions are concentrated on the exit side of the rolled piece. This is because the small thickness of the composite foil causes severe heat loss during rolling, which deteriorates the plasticity of the magnesium alloy on the exit side. Consequently, the damage value on the exit side of the foil is more likely to exceed the critical fracture damage value of the material, ultimately triggering edge cracking.

### 3.2. Rolling Experiment of Mg/Al Composite Foil

**Figure 12:**
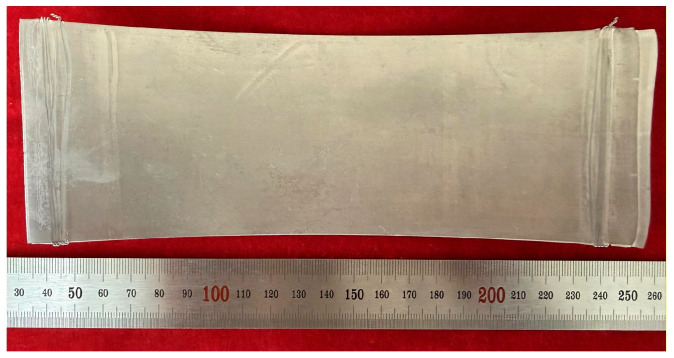
Macroscopic morphology of Mg/Al composite foil after composite pass.

Based on the finite element simulation results presented above, the optimal rolling process parameters for the composite pass were determined as follows: a holding temperature of 400 °C and a reduction ratio of 35%. The Mg/Al composite foils were fabricated via the composite pass using the aforementioned parameters, as shown in Figure 12. It can be observed that the foils prepared under these process parameters exhibit excellent bonding quality and superior surface quality; thus, these parameters were designated as the final process parameters for the composite pass.

**Figure 13:**
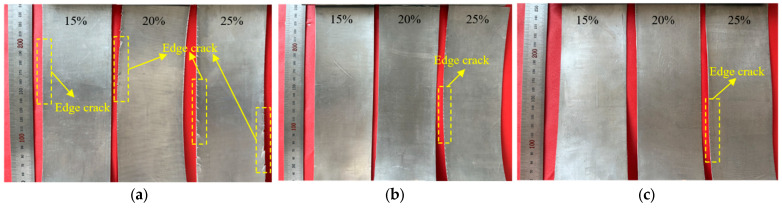
Macroscopic morphology after the second rolling pass: (**a**) 250 °C; (**b**) 300 °C; (**c**) 350 °C.

**Figure 14:**
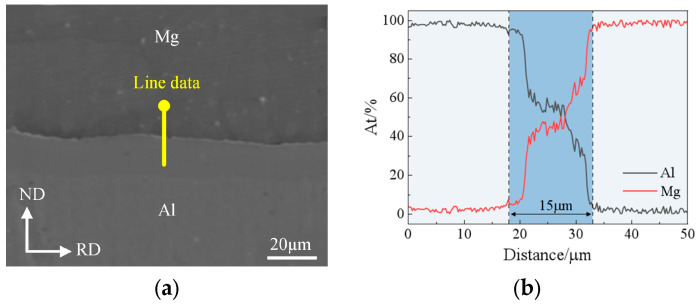
SEM image and EDS line scan curve of the interface after rolling at a holding temperature of 350 °C: (**a**) SEM image; (**b**) EDS line scan curve.

After heating for 3 min at holding temperatures of 250 °C, 300 °C and 350 °C respectively, the composite foils were subjected to the second rolling pass to determine the rolling temperature and reduction ratio for the thinning pass. Figure 13 shows the macro-morphology of Mg/Al composite foils under different rolling temperatures and reduction ratios after the second rolling pass. It can be seen from the figure that edge cracking occurred on the foils at all the aforementioned rolling temperatures when the reduction ratio reached 25%, which is attributed to the poor plasticity of magnesium alloys at low rolling temperatures. The degree of edge cracking was relatively minor at 350 °C; however, after rolling to a thickness of 150 μm at a holding temperature of 350 °C, an EDS line scan was conducted on the bonding interface, which revealed that the scan curve exhibited an “H” shape with a diffusion platform present. This indicates the formation of a relatively thick intermetallic compound layer (Intermetallic Compounds, IMCs) at the bonding interface, as shown in the blue shaded region of Figure 14. Owing to the hard and brittle characteristics of IMCs, which severely impair the secondary formability of the foils, their formation should be avoided in the rolling process. Therefore, a rolling temperature of 300 °C is deemed suitable for the thinning pass, and a reduction ratio of 20% is selected for the corresponding second rolling pass.

**Figure 15:**
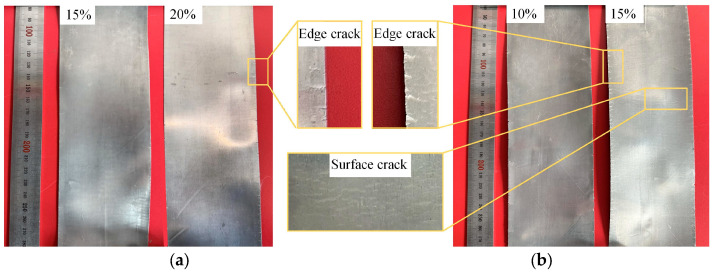
Macroscopic morphology of Mg/Al composite foil after hot rolling passes: (**a**) fourth pass; (**b**) ninth pass.

When rolling to the third pass at a rolling temperature of 300 °C with a 20% reduction ratio, the thickness of the foil had decreased to below 500 μm, and the heating time was further reduced to 2 min at this point. Edge cracking reoccurred during the fourth pass of hot rolling. The reduction ratio was then further lowered to 15% while maintaining the rolling temperature at 300 °C. As shown in Figure 15a, the edge cracking of the foil rolled at a 15% reduction ratio was obviously improved compared with that at a 20% reduction ratio. Although a small amount of edge cracking still occurred, a further decrease in the reduction ratio would lead to a significant increase in the number of rolling passes. Owing to the need for heat preservation and rolling operations between passes, an increase in the number of passes would exert an adverse effect on the bonding interface and result in a substantial rise in energy consumption. Therefore, a 15% reduction ratio was adopted for the subsequent rolling passes. At the ninth rolling pass, the thickness of the foil was reduced to 200 μm, and the heating time was further shortened to 1 min. This heating duration was sufficient to raise the workpiece temperature to the preset value and satisfy the heating requirements. During rolling at this pass, problems, including a significant temperature drop and an aggravated impact of surface defects on the rolling rolls, arose. Continuing to adopt a 15% reduction ratio under this condition would cause the propagation of edge cracks. In addition, the foil strip featured a small thickness at this stage and cooled rapidly after contacting the rolling rolls, which impaired the material’s plasticity and further induced surface cracks. This phenomenon was particularly prominent at the exit end of the foil strip, as shown in Figure 15b. From the perspective of heat transfer mechanism, the foil strip has a large specific surface area; at the moment of contact between the foil strip and the rolling rolls, heat is rapidly transferred from the foil strip to the rolls, generating a rapid cooling effect that exerts a crucial influence on the microstructure and mechanical properties of the magnesium alloy. Therefore, the reduction ratio for this pass was adjusted down to 10%, with the rolling temperature kept constant at 300 °C.

**Figure 16:**
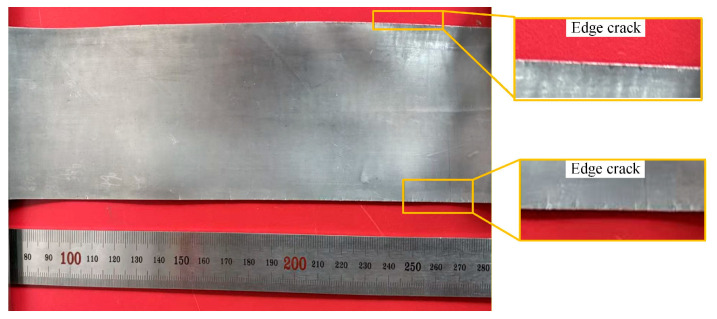
Macroscopic morphology of 150 μm thick Mg/Al composite foil.

After eleven rolling passes, the thickness of the magnesium/aluminum composite foil was reduced to 150 μm, and the foil exhibited an excellent surface quality with slight edge cracks at this stage, as shown in Figure 16. Edge cracking induced by the temperature drop during the hot rolling thinning stage essentially stems from the formation of temperature gradients along RD and TD in the workpiece. These gradients lead to stress concentration and heterogeneous grain deformation behaviors in the edge regions, particularly at the workpiece exit end, thereby initiating crack nucleation. This phenomenon is closely related to the intrinsic characteristics of magnesium alloys, namely their poor high-temperature plasticity and high sensitivity to temperature fluctuations. The rapid temperature drop ultimately results in stress concentration at the exit end and the subsequent formation of edge cracks. Especially for multi-pass hot rolling, the temperature at the workpiece inlet end remains consistently higher than that at the exit end throughout the rolling process. After repeated rolling passes, the differences in microstructure and mechanical properties between the two ends become more pronounced. Therefore, strengthening the thermal insulation measures for the workpiece during rolling is a core factor in the production of hot-rolled foils, which provides clear mechanistic support for subsequent process optimization.

**Figure 17:**
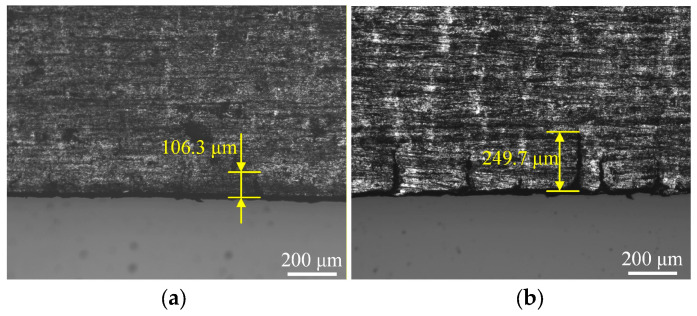
Edge morphology of Mg/Al composite foils under different reduction ratios during cold rolling pass: (**a**) 15%; (**b**) 20%.

When the thickness of the Mg/Al composite foil was reduced to 150 μm, an attempt was made to further reduce the foil thickness by decreasing the roll gap on a two-high rolling mill. However, it was found that further roll gap reduction failed to achieve additional thickness reduction in the foil, indicating that the minimum rollable thickness of the equipment had been reached. Thus, a four-high precision rolling mill was adopted for the subsequent rolling process. As shown in the previous experiments, the Mg/Al composite foil subjected to annealing treatment at 300 °C exhibited excellent plasticity without the formation of intermetallic compounds. Therefore, a short-time annealing treatment was conducted prior to the subsequent rolling to restore the plasticity of the rolled piece and eliminate residual stresses. The annealing parameters were set as follows: an annealing temperature of 300 °C and an annealing time of 5 min. After annealing, rolling was performed with reduction ratios of 15% and 20%, respectively. Figure 17 presents the edge morphology of the Mg side of the foil after the twelfth rolling pass under different rolling reduction ratios. The results in the figure show that only a single edge crack was observed in this region at a 15% reduction ratio, with a crack depth of merely 106.3 μm. In contrast, the foil rolled at a 20% reduction ratio suffered from severe edge cracking, with a crack density of 5.2 cm^−1^ and a maximum crack depth of 249.7 μm.

**Figure 18:**
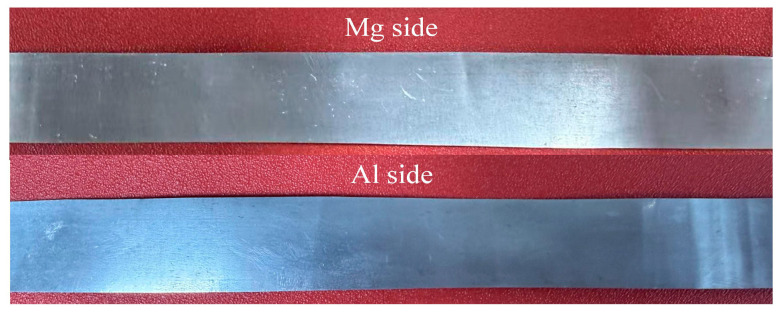
Macroscopic morphology of 30 μm thick Mg/Al composite foil.

In contrast to the hot rolling thinning passes, the reduction ratio was instead increased during cold rolling thinning, which is mainly attributed to the higher roll precision, superior roll surface flatness and more uniform rolling force distribution of the four-high precision rolling mill. After conducting the pre-rolling annealing treatment at 300 °C for 5 min, cold rolling thinning was carried out for 10 passes with a 15% reduction ratio, and the foil was successfully thinned to 30 μm, achieving the target thickness. The macroscopic morphology of the rolled Mg/Al composite foil is presented in Figure 18. As can be seen from the figure, the Mg/Al composite foil rolled by this process exhibited excellent surface quality overall, retained metallic luster and was free of edge cracking.

Hence, it can be concluded that distributing severe plastic deformation across the multi-pass rolling process via multi-pass rolling mitigates the degree of edge cracking of the foil to a certain extent. On the theoretical level, an in-depth investigation into the influence mechanism of the size effect when the foil thickness decreases to a specific range is still required. This effect may stem from the variation in the ratio of the interfacial layer thickness to the total foil thickness and the change in the matching relationship between grain size and foil thickness, which in turn impinges on the forming quality of the composite foil. The rolling process parameters of the Mg/Al composite foil are listed in Table 2.

### 3.3. Interfacial Micromorphology of Mg/Al Composite Foil

**Figure 19:**
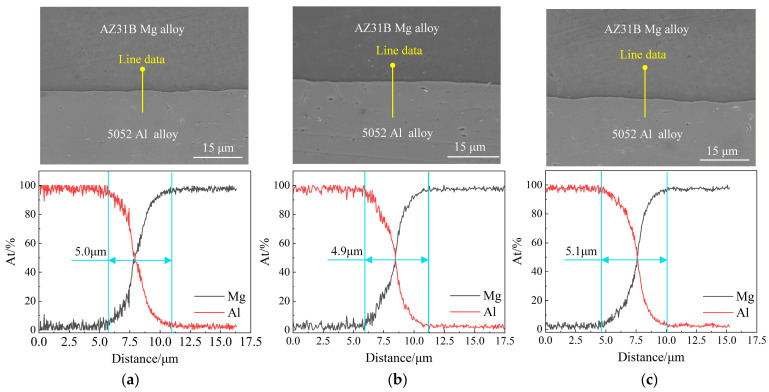

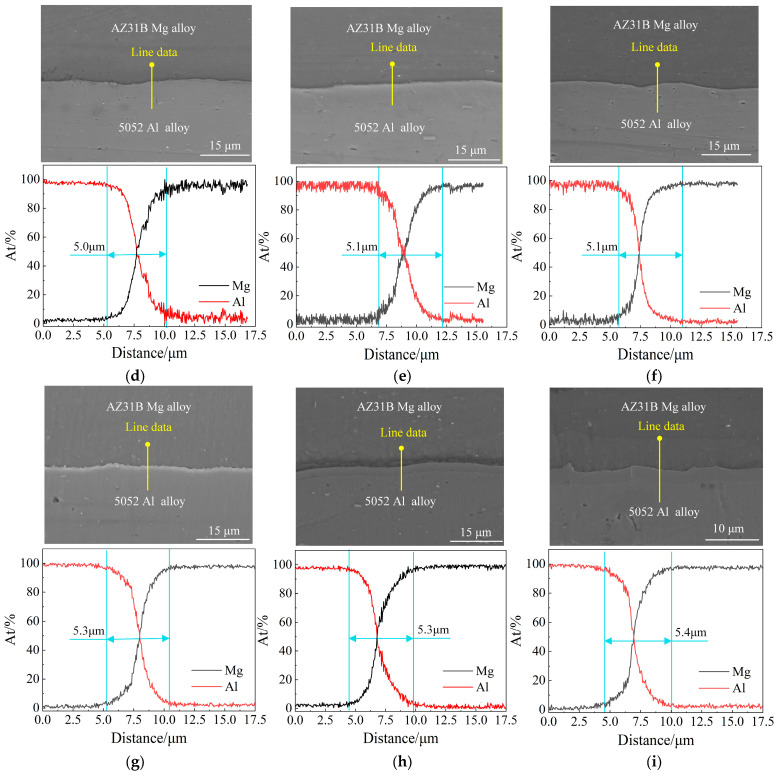
SEM images and EDS line scan results of the bonding interface of the Mg/Al composite foil at different pass: (**a**) first pass; (**b**) second pass; (**c**) fourth pass; (**d**) sixth pass; (**e**) ninth pass; (**f**) eleventh pass; (**g**) twelfth pass; (**h**) seventeenth pass; (**i**) twenty-first pass.

Sampling was conducted on the central region of the sheet at different rolling passes during the rolling process to investigate the effect of multi-pass rolling on the bonding interface of the Mg/Al composite foil. The to-be-detected surfaces of the samples were successively ground with a series of sandpapers by gradation, followed by polishing with an automatic polisher using a polishing agent with an abrasive particle size of 0.5 μm until a scratch-free mirror finish was achieved. The bonding interface was then observed via an SEM. Figure 19 presents the SEM images and EDS line scan results of the bonding interface of the Mg/Al composite foil at different rolling passes.

The SEM images reveal that no defects, such as cracks or interfacial delamination, were observed at the bonding interface of the foil at all passes, and the bonding interface gradually became tortuous with the progress of multi-pass rolling due to the non-uniform distribution of rolling force. The EDS line scan results indicate that the line scan profiles of the foil all exhibited an “X” shape during multi-pass rolling, with the magnesium content increasing gradually and the aluminum content decreasing gradually along the bonding interface, which verifies the formation of an intermediate diffusion layer at the bonding interface [22]. In addition, no step-like profiles were observed for all curves, demonstrating the absence of intermetallic compound (IMC) formation at the bonding interface. Under the conditions of a holding temperature of 400 °C, a holding time of 10 min and a reduction ratio of 35%, the thickness of the diffusion layer of the foil after the composite pass was approximately 5.0 μm (see Figure 19a), indicating excellent bonding quality. During the multi-pass hot rolling thinning process, the thickness of the intermediate diffusion layer exhibited little variation, concentrating at approximately 5.0 μm. During the cold rolling process, the thickness of the intermediate diffusion layer increased and stabilized at approximately 5.3 μm due to the extended annealing time compared with that in hot rolling. After 21 rolling passes, the foil thickness was reduced to 30 μm, with the intermediate diffusion layer thickness reaching 5.4 μm at this stage (see Figure 19i).

This indicates that the rolling result of the composite pass exerts a significant influence on the thickness of the intermediate diffusion layer, and the bonding quality of the Mg/Al composite foil is mainly dependent on the composite pass. Furthermore, short-time holding can impart good plasticity to the foil and effectively inhibit the formation of IMCs.

### 3.4. Metallographic Microstructure Analysis of Mg/Al Composite Foil

**Figure 20:**
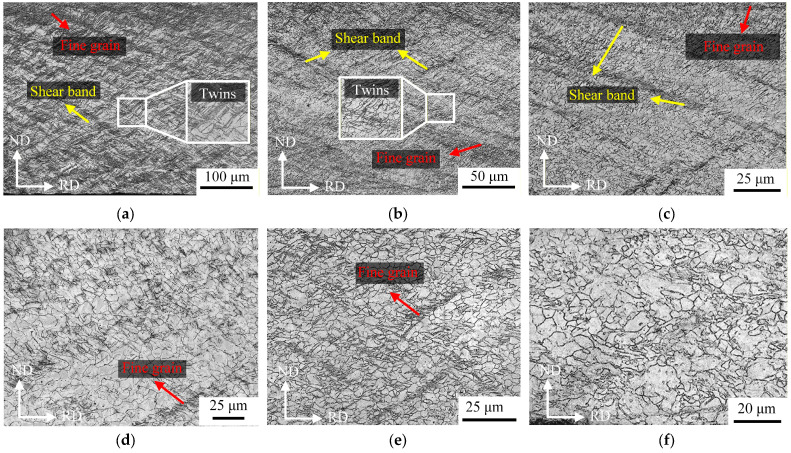
Grain structure of the magnesium matrix in Mg/Al composite foil strip during hot rolling pass: (**a**) first pass; (**b**) second pass; (**c**) fourth pass; (**d**) sixth pass; (**e**) ninth pass; (**f**) eleventh pass.

To investigate the metallographic microstructural evolution of Mg/Al composite foil during multi-pass rolling, samples at different rolling passes were sampled and characterized in this study. After polishing, the polished surfaces were etched with a self-prepared etchant. The microstructures of the magnesium alloy edge at different passes are presented in Figure 20.

After rolling in the composite pass, a large number of shear bands were observed in the near-surface region (see Figure 20a), and a dense fine-grained structure was formed in this region, as well as in the magnesium near-surface region simultaneously. Under the condition of severe plastic deformation, the accumulation of high-density dislocations provides sufficient strain energy storage for grain refinement, which promotes the fracture of original grains [23]. Shear bands induce strain localization, act as the preferential paths for crack initiation and propagation, accelerate fracture, and thus significantly deteriorate the plasticity of magnesium alloys [24,25]. The grain structure of the magnesium alloy after the second rolling pass is shown in Figure 20b, where the density of shear bands decreased significantly. Compared with the composite pass, the microstructural inhomogeneity in different regions of the magnesium alloy was reduced remarkably after the second pass. Inhomogeneous microstructure leads to a significant decrease in the plasticity of magnesium alloys [26]. The grain structure of the magnesium alloy after the fourth rolling pass is displayed in Figure 20c; after multi-pass heat treatment and low-reduction rolling, the density of shear bands in the magnesium alloy structure was obviously lower than that in the early stages, and a large number of equiaxed grains were observed to distribute in the grain boundary region. This microstructural evolution is attributed to the fact that multi-pass low-reduction rolling causes the fracture of a small number of magnesium alloy grains, while multi-pass heat treatment enables complete dynamic recrystallization of some grains [27].

The grain structures after the sixth and ninth rolling passes are shown in Figure 20d,e, respectively. After the sixth and ninth passes, the microstructure of the magnesium alloy was dominated by equiaxed grains, with a small amount of small grains without complete dynamic recrystallization. A small number of shear bands were observed after the ninth rolling pass, yet both their width and length were small. After the eleventh rolling pass, the microstructure of the magnesium alloy exhibited typical equiaxed grain characteristics, and no shear band structure was detected, while the grain size remained inhomogeneous (see Figure 20f).

**Figure 21:**
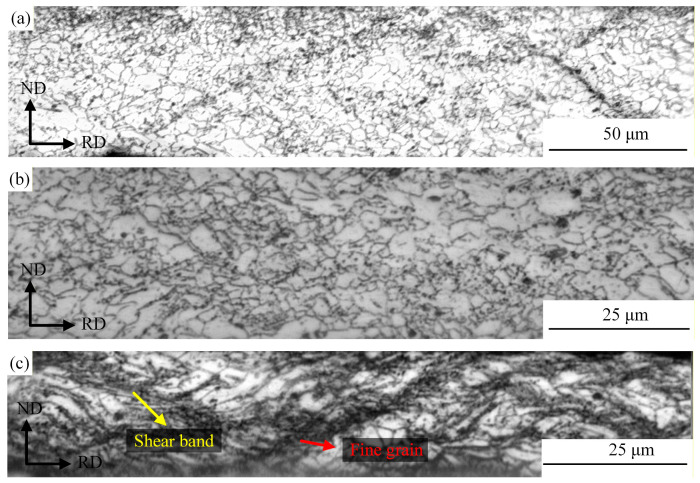
Grain structure of the magnesium matrix in Mg/Al composite foil strip during cold rolling passes: (**a**) twelfth pass; (**b**) seventeenth pass; (**c**) twenty-first pass.

The metallographic microstructures of the magnesium alloy during cold rolling passes are presented in Figure 21. As can be seen from the figure, with the increase in rolling passes and the continuous accumulation of deformation, the grains of the magnesium alloy transformed from uniform deformation to localized deformation [28].

At the twelfth rolling pass (i.e., the first cold rolling pass), the reduction ratio was only 15%. Owing to the small deformation of the material, most grains were equiaxed with relatively intact morphologies, and only a small number of grains elongated along RD were observed with excellent microstructural homogeneity, as shown in Figure 21a. This indicates that the stress–strain distribution was relatively gentle during the rolling of this pass, without the occurrence of severe plastic deformation. After five cold rolling passes, the cumulative deformation induced by multi-pass rolling became prominent. The grain structure underwent severe plastic deformation under the repeated action of rolling force [29]; compared with that at the Twelfth rolling pass, the grains were significantly refined and elongated along the RD, as shown in Figure 21b. At this stage, the inhomogeneity of grain size increased.

After 21 rolling passes, the cumulative cold rolling deformation reached 80%. With a further increase in deformation, the dislocation density rose sharply. Basal slip failed to accommodate the strain caused by severe deformation, leading to stress concentration in localized regions and the subsequent formation of shear bands [30]. Under the intense shearing action inside the shear bands, a large number of fine grains were generated. Large-sized grains were still observed in the figure, yet most of them exhibited an elongated strip-like morphology, as shown in Figure 21c.

## 4. Conclusions

(1) In view of the rolling edge cracking issue of Mg/Al composite foil, this study adopted a hot rolling–cold rolling combined method and comprehensively considered process parameters such as rolling temperature and rolling reduction ratio to investigate the rolling process of Mg/Al composite foil. Through 21 rolling passes, a 30 μm thick composite foil was successfully prepared. This foil featured excellent surface quality, no obvious edge cracking, and favorable bonding at the Mg/Al interface, thus providing reliable process support for the high-quality preparation of Mg/Al composite foil.

(2) The effects of rolling temperature and reduction ratio on edge cracking of Mg/Al composite foil during the composite pass were investigated based on the Cockcroft & Latham fracture criterion. The results show that edge cracking is dominated by tensile stress, and rolling temperature and reduction ratio affect edge cracking by regulating the tensile stress distribution; the damage value at the edge of the rolled piece is significantly higher than that at the central region, and the exit side is the most prone to exceeding the critical fracture value. A comprehensive determination indicates that the optimal process parameters for the composite pass are a rolling temperature of 400 °C and a reduction ratio of 35%, which can effectively inhibit the occurrence of edge cracking. For the hot rolling thinning passes, short-time heating at 300 °C was adopted, and short-time annealing was conducted after the cold rolling passes. This process not only improves the plasticity of the foil but also effectively inhibits the formation of IMCs.

(3) Microscopic analysis reveals that the bonding interface gradually becomes tortuous with the increase in rolling passes, and the thickness of the diffusion layer varies at different stages: the thickness of the diffusion layer remains basically unchanged during the hot rolling stage, while it increases with the increase in rolling passes during the cold rolling stage, eventually forming a 5.4 μm diffusion layer at the bonding interface. With the increase in rolling passes and the accumulation of deformation, the metallographic microstructure of the magnesium alloy exhibits a regular evolution. Multi-pass hot rolling leads to a gradual decrease in the density of shear bands, and dynamic recrystallization results in a microstructure dominated by equiaxed grains; with the increase in cold rolling passes and cumulative deformation, the grains are refined and elongated along the rolling direction, and the elevated dislocation density induces the formation of shear bands and fine grains, leading to inhomogeneous microstructure distribution.

## Figures and Tables

**Table 1:** Chemical composition of AZ31B magnesium alloy and 5052 aluminum alloy.

Material	Si	Fe	Cu	Mn	Mg	Cr	Zn	Al
AZ31B Mg alloy	—	—	0.01	0.32	Others	—	0.9	3.1
5052 Al alloy	0.09	0.24	0.01	0.06	2.43	0.17	—	Others

— indicates zero content of this element.

**Table 2:** Mg/Al composite foil rolling forming process.

Rolling Method	Rolling Pass	Heating Temperature	Reduction Ratio
Hot rolling	1	400 °C	35%
	2–4	300 °C	20%
	5–9	300 °C	15%
	10–11	300 °C	10%
Cold rolling	12–21	—	15%

— indicates no heating, with cold rolling performed at room temperature.

## Data Availability

The original contributions presented in this study are included in the article. Further inquiries can be directed to the corresponding author.
